# Deep Learning-Based Automated Detection of Arterial Vessel Wall and Plaque on Magnetic Resonance Vessel Wall Images

**DOI:** 10.3389/fnins.2022.888814

**Published:** 2022-06-01

**Authors:** Wenjing Xu, Xiong Yang, Yikang Li, Guihua Jiang, Sen Jia, Zhenhuan Gong, Yufei Mao, Shuheng Zhang, Yanqun Teng, Jiayu Zhu, Qiang He, Liwen Wan, Dong Liang, Ye Li, Zhanli Hu, Hairong Zheng, Xin Liu, Na Zhang

**Affiliations:** ^1^Paul C. Lauterbur Research Center for Biomedical Imaging, Shenzhen Institute of Advanced Technology, Chinese Academy of Sciences, Shenzhen, China; ^2^Faculty of Information Technology, Beijing University of Technology, Beijing, China; ^3^United Imaging Healthcare Co., Ltd., Shanghai, China; ^4^Department of Computing, Imperial College London, London, United Kingdom; ^5^Department of Radiology, Guangdong Second Provincial General Hospital, Guangzhou, China

**Keywords:** deep learning, MR vessel wall imaging, automatic segmentation, plaques, automated detection

## Abstract

**Purpose:**

To develop and evaluate an automatic segmentation method of arterial vessel walls and plaques, which is beneficial for facilitating the arterial morphological quantification in magnetic resonance vessel wall imaging (MRVWI).

**Methods:**

MRVWI images acquired from 124 patients with atherosclerotic plaques were included. A convolutional neural network-based deep learning model, namely VWISegNet, was used to extract the features from MRVWI images and calculate the category of each pixel to facilitate the segmentation of vessel wall. Two-dimensional (2D) cross-sectional slices reconstructed from all plaques and 7 main arterial segments of 115 patients were used to build and optimize the deep learning model. The model performance was evaluated on the remaining nine-patient test set using the Dice similarity coefficient (DSC) and average surface distance (ASD).

**Results:**

The proposed automatic segmentation method demonstrated satisfactory agreement with the manual method, with DSCs of 93.8% for lumen contours and 86.0% for outer wall contours, which were higher than those obtained from the traditional U-Net, Attention U-Net, and Inception U-Net on the same nine-subject test set. And all the ASD values were less than 0.198 mm. The Bland–Altman plots and scatter plots also showed that there was a good agreement between the methods. All intraclass correlation coefficient values between the automatic method and manual method were greater than 0.780, and greater than that between two manual reads.

**Conclusion:**

The proposed deep learning-based automatic segmentation method achieved good consistency with the manual methods in the segmentation of arterial vessel wall and plaque and is even more accurate than manual results, hence improved the convenience of arterial morphological quantification.

## Introduction

Confirming and risk stratifying vulnerable plaques is especially important for the clinical prevention and treatment of ischemic stroke. Magnetic resonance vessel wall imaging (MRVWI) can directly visualize arterial vessel walls and characterize vulnerable plaques. It has been widely used as an emerging non-invasive imaging modality for evaluating and identifying patients at risk for ischemic stroke (Loewe et al., 1998; Frank, 2001; Burtea et al., 2012; Dieleman et al., 2014).

Quantitative morphologic measurements of the arterial vessel wall and plaques based on MRVWI have been proven to have good reproducibility (Mandell et al., 2017; Saba et al., 2018; Zhang et al., 2018) and suggested to be imaging markers to monitor the progression and regression of ischemic stroke during medical management or drug development (Adams et al., 2004; Minarro-Gimenez et al., 2018). However, quantitative measurements are currently of limited use in clinical practice because manual segmentation of the vessel wall and plaque is labor intensive and requires continuous training of personnel (Qiao et al., 2011). It usually takes a trained expert more than 30 min to analyze MRVWI images of one patient from the manual reconstruction of two-dimensional (2D) slices to the manual segmentation of the vessel wall and plaque. In addition, the main challenge of MRVWI-based segmentation is the low contrast between the vessel wall and the surrounding tissues, which causes the accuracy of segmentation to depend heavily on the knowledge and experience of experts.

Over the past years, several studies have used computer-aided diagnosis to improve the efficiency and accuracy of segmentation and reduce the burden on doctors for the interpretation of medical images (Ladak et al., 1999; Sakellarios et al., 2012; Jodas et al., 2018). However, these methods sometimes require user intervention. With the widespread application of artificial intelligence in the field of medical image analysis, convolutional neural networks (CNNs) have achieved important breakthroughs in image segmentation tasks (Anwar et al., 2018; Yamashita et al., 2018; Maier et al., 2019; Dutta et al., 2020; Ohsaka, 2020; Taghanaki et al., 2021). Compared with traditional automatic segmentation methods, CNNs can automatically learn abundant image features to achieve fast and more accurate segmentation. Some studies have used CNN to achieve carotid arterial vessel segmentation (Tsakanikas et al., 2020; Zhu et al., 2021). In addition, some other studies used CNN to segment carotid arterial vessel wall. Among them, Chen et al., 2019 developed tractlet refinement and polar transformation for carotid artery localization and vessel wall segmentation and achieved high accuracy (Chen et al., 2019). Samber et al. (2020) used CNN to the task of delineating carotid vessel walls based on 2D T2-weighted MRVWI images. However, all these studies are aimed at the segmentation of carotid arterial vessels or vessel walls. There is a paucity of study on the automatic segmentation of intracranial arterial vessel wall. Recently, Shi et al. (2019) made a preliminary attempt to automatically segment the intracranial arterial vessel wall using a U-Net-like fully convolutional network based on whole-brain MRVWI images of 56 patients. As we know, atherosclerosis is a diffuse disease that can occur in any artery. It is more important and clinically significant to estimate the effect of a segmentation model based on MRVWI images including more arteries (intracranial and carotid arteries) in a larger patient population.

In this study, a fully automated method for the segmentation of the arterial lumen and vessel wall based on intra- and extracranial MRVWI images was developed and evaluated in a large cohort of patients with ischemic stroke.

## Materials and Methods

### Study Population

The prospective study was approved by the local institutional review board, and all patients gave the informed consent. From January 2019 to April 2020, 129 consecutive patients (age range 46–78 years, mean age 58.6 ± 18.9 years) requiring high-resolution MRVWI scans in 3 centers were recruited for the study.

### Image Acquisition

All MRVWI images were acquired using a T1-weighted 3D-variable flip-angle fast spin-echo (FSE) sequence, namely MATRIX (Modulated flip Angle Technique in Refocused Imaging with extended echo train) on a 3T whole-body MR system (uMR780, United Imaging Healthcare Co., Ltd., Shanghai, China). The imaging parameters were as follows: sagittal imaging orientation, repetition time (TR)/echo time (TE) = 800/13.92 ms, field of view = 230 mm × 192 mm × 154 mm, matrix size = 384 × 320 × 256, spatial resolution = 0.6 mm × 0.6 mm × 0.6 mm without interpolation, echo train length = 46, receiver bandwidth = 600 Hz/pixel, compress sensing-based acceleration rate (uCS) = 5.2, scan time = 4 min and 49 s. The study was approved by the local institutional review board, and informed consent was waived for the retrospective study.

### Image Preprocessing

**FIGURE 1:**
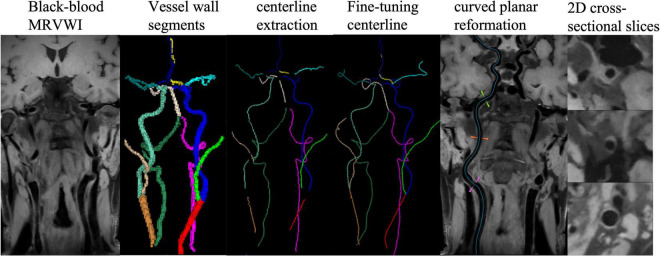
The workflow for proposed centerline extraction of intracranial and carotid artery.

A dedicated plaque analysis software (uWS PlaqueTool, United Imaging Healthcare Co., Ltd., Shanghai, China) was used for image preprocessing. First, curved-planar reconstruction for all intracranial and carotid arterial segments were automatically performed using centerline extraction algorithm. Then, 2D cross-sectional slices were reconstructed for all plaques and seven main arterial segments: the common carotid artery (CCA), the internal carotid artery (ICA) and bifurcation, the anterior cerebral artery (ACA), the middle cerebral artery (MCA), the basilar artery (BA), the vertebral artery (VA), and the posterior cerebral artery (PCA), and manually delineated the lumen and outer wall contours by five experienced radiologists with more than 6 years of experience. Representative images processed with the automatic workflow are shown in Figure 1.

Five patients were excluded from training database due to the poor image quality with motion artifacts. A total of 13,962 2D MRVWI slices were reconstructed from 7 arterial segments of 124 patients, of which, 9,073 slices and 3,889 slices reconstructed from 115 patients were used as the training and validation sets to build and optimize the model. In total, 1,000 slices reconstructed from the remaining 9 patients were used as the test set to evaluate the obtained model. These slices of the nine patients were manually delineated twice to compare the results of the deep learning CNN-based approach with the variability of the data between two manual reads to see if the variance between the CNN and ground truth was within the variability of assessment of expert readers.

For most slices derived from normal arterial segments or segments with slightly thickened vessel walls, an ellipse tracing tool composed of four coordinate points is used for quick delineation. For some slices with irregular shapes of the lumen and vessel wall caused by large and complex plaques, a free-shape tracing tool composed of multiple coordinate points is used for more accurate delineation. Due to the large amount of data to be labeled, five readers independently performed the above delineation on different data and cross-checked the delineation results to ensure that each slice was delineated by at least two readers by consensus. When there is discrepancy between the labeling and checking readers, a third senior reader was invited for the final decision by consensus. To avoid model overfitting, the training dataset was expanded by nearly six times from 9,073 to 54,438 slices through rotation, translation, and padding. Then, each slice was interpolated to 0.075 mm × 0.075 mm for reducing the morphologic measurement error and resized to 256 × 256 pixels and grayscale normalized as follows to reduce the inconsistent characteristics of the images.


(1) $$\text{norm}=\left(\frac{x_{i}-x_{\text{min}}}{x_{\text{max}}-x_{\text{min}}}\times \left(x_{\text{maxv}}-x_{\text{minv}}\right)+x_{\text{minv}}\right)$$

where *x*_*i*_ denotes the pixel value, and *x*_*minv*_ and *x*_*maxv*_ represent upper and lower bounds of normalized. Here, *x*_minv_ = 0,*x*_maxv_ = 1.

In summary, the proposed model was trained on 54,438 2D MRVWI slices, validated on 3,889 slices, and tested on 1,000 slices.

### Vessel Wall Segmentation

**FIGURE 2:**
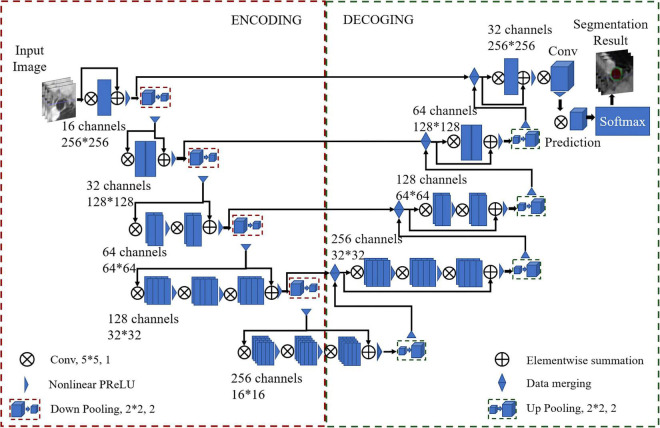
The architecture of the proposed CNN-based VWISegNet.

A U-Net-like (Ronneberger et al., 2015) multiclass deep learning architecture was proposed to segment the vessel wall and lumen, named VWISegNet. The main architecture of the network is shown in Figure 2. It consists of an encoder path and an asymmetric decoder path followed by a pixelwise classifier that enables precise pixel classification. The two branches were connected by a skip connection. A filter with a size of 5 × 5 and stride of 1 was applied to all the convolutional layers to extract fine features from the resized images. Compared with the traditional U-Net network, the VWISegNet has more residual units, and these residual units can better propagate information between the low and high levels, alleviating vanishing gradient problem and allowing the network to obtain better results.

A convolutional layer with a *K* = 2 kernel and stride of 2 is used for downsampling as a substitute for the pooling layer in the encoder path. Instead of directly downsampling, each layer adds a residual unit that achieves fast convergence and better network performance. Utilizing a 1 × 1 kernel size with a stride of 1 subsequently performed beyond the last stage to generate outputs of the same size as the input images. In the process of training, convolution kernel size was decided by considering the influence of the perception field and computational efficiency. The 5 × 5 convolution kernel can obtain a larger perception field and can extract image features better. The 20 epochs are run at a learning rate of 3e−5 with a batch size of 32. With 20 epochs of training, our network model was sufficiently converged and very stable. The Dice loss function is selected for calculating the loss of the lumen and the outer wall. Adaptive moment estimation (Adam) is used to optimize the model with a momentum of 0.9. The parametric rectified linear units (PReLU) function is used as the activation function. PReLU avoids the “dead features” problem caused by zero gradient. In this article, the filter size of 2 × 2 is the common size in the upsampling and downsampling.

Since traditional U-Net is some sort of standard for medical image segmentation, the proposed VWISegNet was compared with respect to the performance to this benchmark and recently published Attention U-Net, and Inception U-Net on the same test data. In addition, the VWISegNet was compared with champion group results of the Carotid Artery Vessel Wall Segmentation Challenge^1^ held MICCAI 2021 and SMRA 2021 on their dataset.

### Evaluation Indicators and Statistical Analysis

The Dice similarity coefficient (DSC) and the average surface distance (ASD) were used to quantitatively evaluate the similarity between automatic and manual segmentation results (Bertels et al., 2019; Eelbode et al., 2020). ASD is obtained by calculating the average of all the distances from each point on the automatic segmentation boundary to the corresponding point on the ground-truth boundary. To evaluate the accuracy of the proposed automatic segmentation method for the estimation of arterial vessel wall morphological parameters, the lumen area, vessel wall area, mean wall thickness, and normalized wall index calculated using the automatic segmentation method were also compared with those calculated using the manual segmentation method.

Statistical analyses were performed using SPSS (version 19.0, NY, United States). The Bland–Altman, scatter plot, and intraclass correlation coefficient (ICC) were used to evaluate the agreement between the automatic and manual methods and between two manual reads for the lumen and vessel wall measurement. An ICC value of less than 0.4 was considered poor agreement, a value of 0.4–0.75 was considered good agreement, and a value of 0.75 or greater was considered excellent agreement.

## Results

**FIGURE 3:**
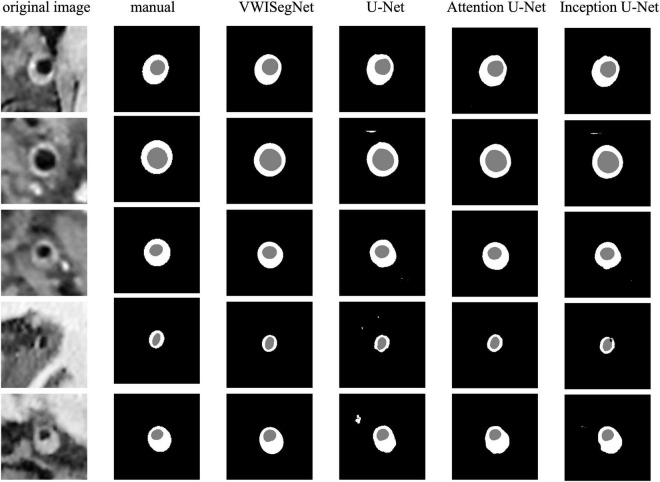
A representative comparison between the proposed VWISegNet and the traditional U-Net, Attention U-Net, and Inception U-Net on the segmentation of vessel wall.

The VWISegNet model converged after 5,000 iterations within 3 epochs. The plots of convergence for both training and validation data are shown in Supplementary Figures 1, 2, respectively. The mean of DSC reached 93.8 ± 6.3% for lumen contours and 86.0 ± 9.0% for outer wall contours on the nine-subject test set. These DSC values were higher than those obtained from the traditional U-Net, Attention U-Net, and Inception U-Net. The segmentation results for lumen contours and vessel wall contours of different methods are summarized in Table 1. Representative results of the proposed VWISegNet, the traditional U-Net, Attention U-Net, and Inception U-Net on the segmentation of vessel wall are shown in Figure 3. The training convergence plot of U-Net, Attention U-Net, and Inception U-Net are shown in Supplementary Figures 3–5, respectively. The ASD of the proposed VWISegNet was 0.068 ± 0.016 and 0.095 ± 0.048 mm for the lumen and the outer wall contours, respectively. The DSC and ASD for the lumen and the vessel wall when comparing the automatic and manual methods on the nine-subject test set are summarized in Table 2. Compared with the Carotid Artery Vessel Wall Segmentation Challenge, VWISegNet achieved the better segmentation performance. The DSC and the difference in lumen area, outer wall area, and normalized wall index measured by VWISegNet and manual method were 78.1 ± 15.2%, 0.063 ± 0.134, 0.065 ± 0.106, and 0.067 ± 0.066, respectively. However, the champion group of the challenge achieved a lower DSC of 77.5 ± 14.5%, and a larger difference in lumen area, outer wall area, and normalized wall index, which were 0.086 ± 0.256, 0.072 ± 0.159, and 0.080 ± 0.071, respectively. The VWISegNet achieved a Hausdorff distance of 0.321 ± 0.852, which was not good as that of 0.246 ± 0.443 achieved by champion group of the challenge.

**TABLE 1:** The segmentation results for lumen contours and vessel wall contours of different methods.

		U-Net (mean ± SD)	VWISegNet (mean ± SD)	Attention U-Net (mean ± SD)	Inception U-Net (mean ± SD)
DSC (%)	Lumen	86.7 ± 16.9	93.8 ± 6.3	92.0 ± 8.5	86.8 ± 17.4
	Vessel wall	73.1 ± 14.6	86.0 ± 9.0	79.7 ± 9.8	74.3 ± 16.4

*DSC, Dice similarity coefficient.*

**TABLE 2:** The DSC and ASD for the lumen and the vessel wall when comparing automatic and manual method on the nine-subject test set.

		Mean ± SD	ACA	CCA	ICA	MCA	BA	PCA	VA
DSC (%)	Lumen	93.8 ± 0.764	93.6	94.7	94.3	93.8	94.1	92.3	94.2
	Vessel wall	86.0 ± 1.866	84.6	88.3	87.7	85.7	87.6	83.9	84.0
ASD (mm)	Lumen	0.068 ± 0.016	0.063	0.064	0.098	0.056	0.060	0.057	0.080
	Vessel wall	0.095 ± 0.048	0.093	0.124	0.198	0.099	0.074	0.053	0.138

*DSC, Dice similarity coefficient; ASD, average surface distance; SD, standard deviation.*

**FIGURE 4:**
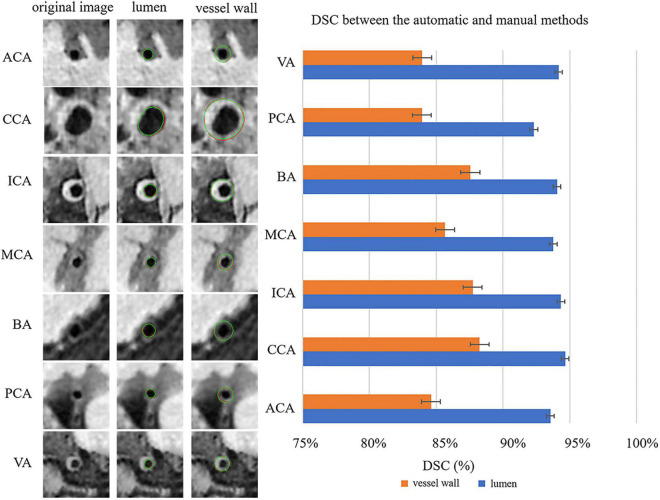
Representative images and DSCs between the automatic and manual methods for the seven arterial segments. On the left, the first column represents the original cross-sectional slices reconstructed from MR vessel wall images; in the second column, the red contours represent the automatic segmentation results of the lumen, and the green contours represent the manual segmentation results of the lumen. In the third column, the red contour represents the automatically segmented results of the outer vessel wall, and the green contour represents the manual segmentation results of the outer vessel wall. The DSCs of the seven arterial segments are shown on the right using a bar plot.

**FIGURE 5:**
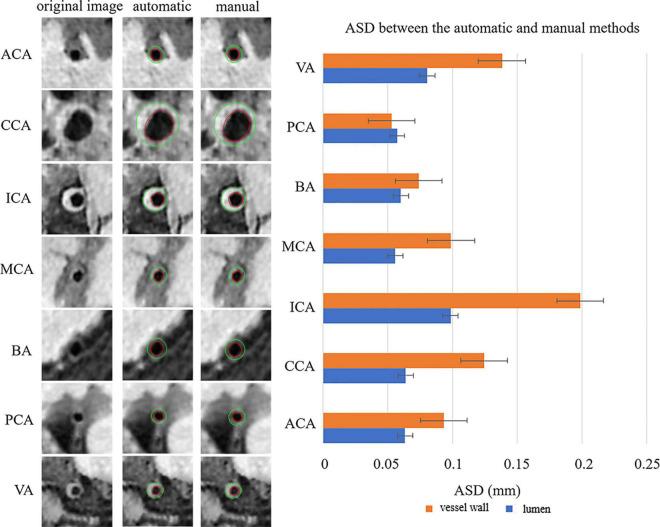
Representative images and the ASDs between the automatic and manual methods for the seven arterial segments. On the left, the first column represents the original cross-sectional slices reconstructed from MR vessel wall images, the second column represents the automatically segmented lumen contour and outer vessel wall contour, and the third column represents the manual segmentation results of lumen and outer vessel wall contours of the seven arterial segments. The red contour represents the lumen, and the green contour represents the outer vessel wall. The ASDs of the seven arterial segments are shown on the right using a bar plot. The standard deviation (SD) represents the amount of dispersion of the variable and is calculated as the root square of the variance. ASD, average surface distance.

**FIGURE 6:**
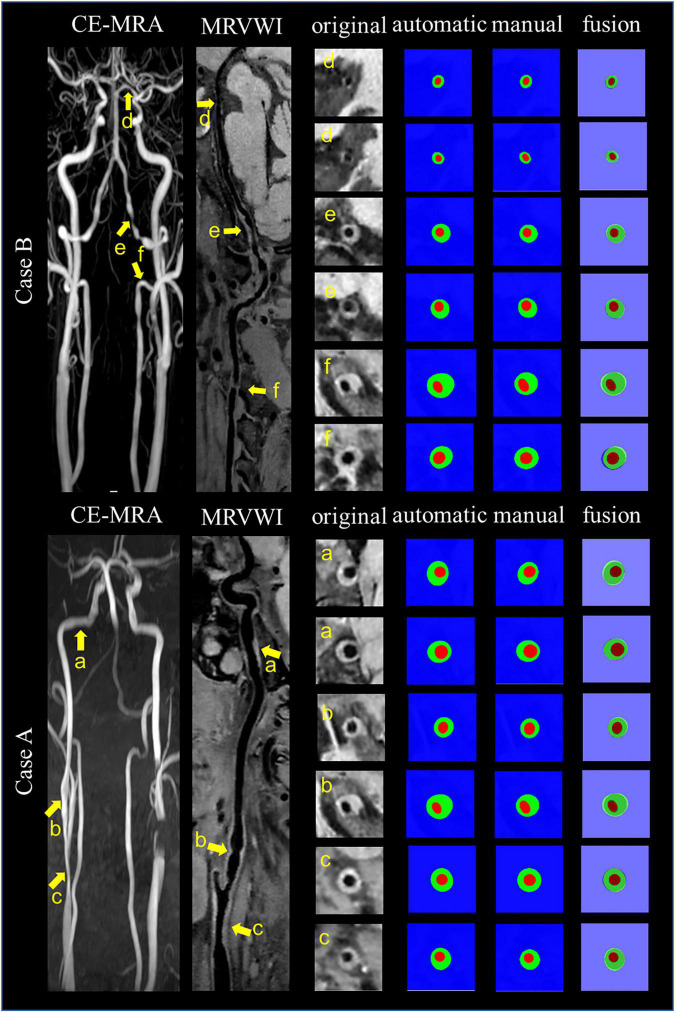
Representative images and segmentation results from two clinical cases. Case A and Case B represent the images with anterior circulation and posterior circulation, respectively. Case A shows three stenoses at the right CCA to ICA (arrows a, b, and c on the CE-MRA image), corresponding plaques (arrows a, b, and c on the MR vessel wall image), original cross-sectional slices reconstructed from the plaques (a–c), the automatic and manual segmentation results for the plaques, and the segmentation results of fusion from left to right. Case B shows three stenoses at the left VA to PCA (arrows d, e, and f on CE-MRA image), corresponding plaques (arrows d, e, and f on MR vessel wall image), original cross-sectional slices reconstructed from the plaques (d–f), the automatic and manual segmentation results for the plaques, and the segmentation results of fusion from left to right.

Figure 4 shows the automatic segmentation DSC results of the lumen and outer vessel wall contours on seven arterial segments and their manual segmentations for reference. All the DSC values were greater than 80%, especially for lumen contour detection, and the DSC values were all greater than 83.9%. The lowest DSC value for the lumen contour was 92.3% of the PCA, and the highest DSC value was 94.7% of the CCA. For the outer wall contour, the lowest and highest DSC values were 83.9% of the PCA and 88.3% of the CCA. In general, a DSC higher than 70.0% is a good segmentation result. Figure 5 shows the automatic segmentation ASD results of the lumen and outer wall contours on seven arterial segments and their manual segmentations for reference. The lowest ASD value for the lumen contour was 0.056 of the MCA. For the outer wall contour, the lowest ASD was 0.053 for PCA. ICA had the highest values for both the lumen and outer wall among the seven segments. The DSC and ASD results indicated that the proposed automatic segmentation method was able to provide a reasonable segmentation result of lumen and outer wall contours. As shown in Figure 6, two representative segmentation results of plaques in the anterior circulation and posterior circulation show visually consistent delineation of the lumen and outer wall contours.

**FIGURE 7:**
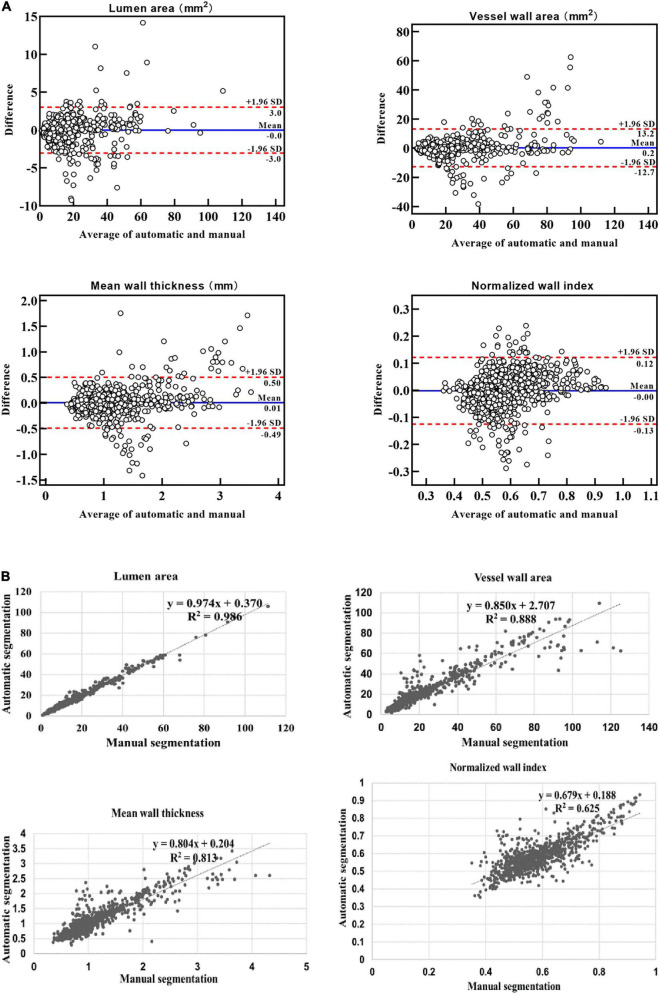
Bland–Altman plots and scatter plots between the automatic method and manual method for the lumen area, wall area, mean wall thickness, and normalized wall index. In panel **(A)**, the blue lines indicate the mean difference, and the red line represents the 95% CI (computed through average difference ±1.96 SD of the difference). CI, confidence intervals; SD, standard deviation. In panel **(B)**, the *x*-axis shows the manual segmentation value. The *y*-axis represents the automatic segmentation value.

The Bland–Altman plots (Giavarina, 2015) for the lumen area, vessel wall area, mean wall thickness, and normalized wall index when comparing the proposed automatic segmentation method with the manual segmentation method are shown in Figure 7A. Random bias scattering patterns between the mean differences were observed. The mean differences between the two methods were −0.002 for the lumen area, 0.246 for the vessel wall area, 0.006 mm for the mean wall thickness, and −0.002 for the normalized wall area, which implied that there was a good agreement with a small bias between the two methods. Figure 7B shows the scatter plots for the four measurements between the automatic segmentation method and the manual segmentation method. The *R*^2^ values of the lumen area, vessel wall area, mean wall thickness, and normalized wall index were 0.986, 0.888, 0.625, and 0.813, respectively.

**FIGURE 8:**
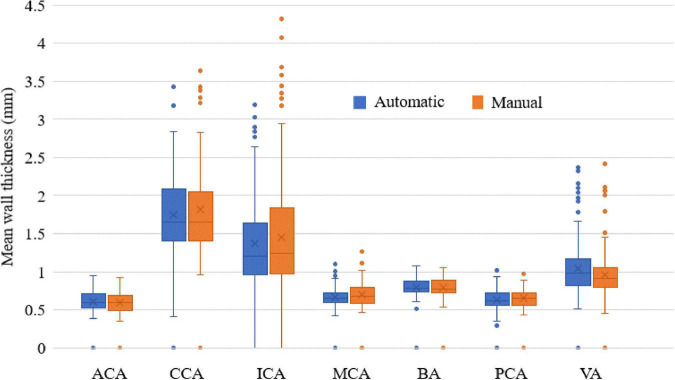
The mean wall thickness of seven arterial segments. Boxplot shows the median with 25th and 75th percentiles. The “×” symbol points the mean and “o” shows the outliers.

In addition, the ICC values between the automatic and manual segmentation methods and between two manual reads for the four measurements are summarized in Table 3. The automatic segmentation method provided an excellent agreement with both manual methods in the measurement of lumen area, vessel wall area, mean wall thickness, and normalized wall index, with all ICC values greater than 0.780. For all the measurements, the ICC values between the automatic segmentation method and the manual method were greater than that between two manual reads. More specifically, the mean wall thickness of seven arterial segments was compared using a boxplot when comparing automatic and manual results. As shown in Figure 8, for each of the seven arterial segments, the mean value difference was not more than 0.100 mm between the automatic and manual segmentation methods.

**TABLE 3:** The agreement of lumen and vessel wall measurements (ICC values with 95% CI) between automatic and manual segmentation methods and between two manual reads.

	Lumen area (mm^2^)	Vessel wall area (mm^2^)	Mean wall thickness (mm)	Normalized wall index
ICC (GT1-X) (95% CI)	0.987 (0.985–0.989)	0.936 (0.928–0.943)	0.896 (0.883–0.907)	0.782 (0.756–0.805)
ICC (GT2-X) (95% CI)	0.988 (0.986–0.989)	0.951 (0.944–0.956)	0.907 (0.896–0.918)	0.797 (0.773–0.819)
ICC (GT1-GT2) (95% CI)	0.987 (0.985–0.988)	0.926 (0.916–0.934)	0.877 (0.862–0.891)	0.704 (0.671–0.734)

*ICC, intraclass correlation coefficient; CI, confidence intervals; ICC (GT1-X), ICC analysis between first manual read and automatic read; ICC (GT2-X), ICC analysis between second manual read and automatic read; ICC (GT1–GT2), ICC analysis between two manual reads.*

## Discussion

A fast and accurate detection method of vessel wall and lumen contours is useful for clinically efficient and accurate quantitative assessment of plaques, which is essential for evaluating plaque progression and treatment effects. In this study, an automatic segmentation approach using a CNN-based deep learning technique was proposed to segment the contour of the vessel wall and lumen on MRVWI images and achieved good-to-excellent agreement with the manual method. With this proposed method, automatic segmentation of the main arterial segments of intracranial and carotid arteries of one subject can be completed in a few minutes. With this potential advancement, it is likely to be used for rapid and accurate quantitative assessment of plaques during MRI scans, thereby assisting in identifying plaques and patients at risk of stroke.

The proposed fully automatic segmentation method achieved DSC larger than 80.0 and 90.0% for the segmentation of outer wall contour and lumen contour, respectively. In particular, the DSC result of the CCA was the best, reaching more than 94.7 and 88.3% for the lumen and the outer wall contours, respectively. The reason may be that CCA is larger than other carotid or intracranial arterial segments—the average diameter of the CCA lumen is 6–7 mm. Therefore, the higher signal-to-noise ratio and contrast-to-noise ratio of the CCA vessel wall facilitate segmentation of the lumen and vessel wall. Compared to previous study with DSC achieved of 88.9 and 76.7% for lumen and vessel wall, respectively (Shi et al., 2019), our automatic segmentation method showed comparatively a better agreement with the manual method. This could be explained by the fact that more training and validation samples and a network structure with more residual units were used in this study. Compared with previous studies for carotid artery segmentation (Chen et al., 2019; Samber et al., 2020), our results are close to but lower than these segmentation results. In addition, the Hausdorff distance of the VWISegNet was not good as the champion group of the Carotid Artery Vessel Wall Segmentation Challenge. The reason maybe that these studies were all only based on carotid artery segmentation, whereas our study is based on the segmentation of both intracranial and carotid arteries. The larger size of carotid artery than intracranial artery was more conducive to segmentation.

All the ASD values are less than 0.198 mm, which also exhibited the good segmentation consistency of the proposed automatic segmentation method with the manual method. And our results are also significantly lower than the previous study by Zhu et al. (2021), which achieved ASD values of 0.682 and 0.960 mm for lumen and vessel wall segmentation, respectively. Generally, a larger DSC value corresponds to a smaller ASD value. Although CCA has the largest DSC values for both the lumen and vessel wall, in our study, the smallest ASD value was found in MCA for the lumen and PCA for the vessel wall. It is supposed that the large contour size of CCA may have caused a larger error, with the MCA and PCA being relatively smaller segments. ICA has the highest ASD value for both the lumen and outer wall among the seven segments, possibly because ICA has the highest probability of plaques, and the highest number of plaques results in poor image quality. Therefore, a larger error result in the highest ASD value of ICA. If the point on the automatic segmentation boundary is overlapped with the corresponding point on the ground-truth boundary, the distance is 0. If the corresponding points on the two boundaries are not overlapped, the distance is a multiple of the interpolation resolution 0.075 mm. Therefore, the average of all the distances may be less than 0.075 mm. The lower value of ASD, the more similar between the automatic segmentation result and the manual result.

In addition, the Bland–Altman plots and scatter plots of lumen area, vessel wall area, mean wall thickness, and normalized wall index also showed a good agreement between the automatic and manual methods. However, the outliers in the Bland–Altman plots indicated that for some small arterial segments (such as the MCA) and some arterial segments with a low contrast-to-noise ratio between the vessel wall and surrounding tissues, the error between automatic segmentation and manual segmentation was relatively larger. For the ICC analysis, ICC values between the automatic segmentation method and each manual method were greater than that between two manual reads. This suggested that the proposed automatic method could not only replace manual method to reduce the workload of the radiologist and increase the convenience but also improved the accuracy of the segmentation results.

Comparing with U-Net, the proposed VWISegNet demonstrated higher DSC for both lumen and vessel wall segmentation and hence the better segmentation performance. This maybe benefit from the more residual units of VWISegNet, which can better extract image features and achieve faster convergence.

There are several potential limitations of this study. First, the sample size of data with plaques is relatively small for deep learning-based segmentation. However, the segmentation of normal arterial vessel walls is the basis for plaque segmentation and recognition. Second, although the dataset was collected from three different centers, they were all acquired with the same protocol. The dataset acquired from different MRI systems is warranted to train a segmentation model in the next work to ensure that the model can be performed on a more diverse multicenter dataset. Third, this study is based on 2D segmentation of what is inherently a 3D problem. The 2D slices are needed to be reconstructed from the acquired 3D MRVW images, and then the segmentation is performed on the 2D slices. In addition, the proposed segmentation method is aimed at the situation where there is only one artery in the 2D slice. However, there may be many different arteries on the 2D slice. It is believed that incorporating 3D context information into the model will make it possible to distinguish different blood vessels and improve the segmentation results. Finally, in the future work, the computer-aided detection (CADe) system for MRVW images is expected to do the detection and segmentation at the same time.

## Conclusion

In conclusion, the proposed deep learning-based high-performing, automatic segmentation method has achieved good consistency with manual methods in terms of arterial morphologic measurements and is even more accurate than manual results, which could potentially be useful for monitoring plaque progression and clinical treatment effects.

## Data Availability Statement

All data generated or analyzed during this study are included in this published article and its Supplementary Material.

## Ethics Statement

The studies involving human participants were reviewed and approved by the Shenzhen Institutes of Advanced Technology, Chinese Academy of Sciences, Shenzhen, China. The patients/participants provided their written informed consent to participate in this study.

## Author Contributions

WX: data acquisition, analysis, training model, interpretation, and drafted the manuscript. XY: data acquisition, analysis, training model, and interpretation. YIL, GJ, SJ, and ZG: data acquisition and analysis. YM, SZ, YT, and JZ: statistical analysis and interpretation of data. QH, LW, and DL: study design, data interpretation, and revise the manuscript. YEL, ZH, HZ, and XL: conception and design of the study. NZ: study design, provided supervision, and critical review of the manuscript. All authors read and approved the final manuscript.

## Conflict of Interest

XY, ZG, YM, SZ, YT, JZ, and QH were employed by United Imaging Healthcare Co., Ltd. The remaining authors declare that the research was conducted in the absence of any commercial or financial relationships that could be construed as a potential conflict of interest.

## Publisher’s Note

All claims expressed in this article are solely those of the authors and do not necessarily represent those of their affiliated organizations, or those of the publisher, the editors and the reviewers. Any product that may be evaluated in this article, or claim that may be made by its manufacturer, is not guaranteed or endorsed by the publisher.

## Footnotes

1
https://vessel-wall-segmentation.grand-challenge.org/

## Supplementary Material

Supplementary Figure 1 The plot of convergence for training data.

Supplementary Figure 2 The plot of convergence for validation data.

Supplementary Figure 3 The training convergence plot of U-Net.

Supplementary Figure 4 The training convergence plot of Attention U-Net.

Supplementary Figure 5 The training convergence plot of Inception U-Net.
