# Supplementary figures and images for: Deep Learning-Based Automated Detection of Arterial Vessel Wall and Plaque on Magnetic Resonance Vessel Wall Images
Source: Front Neurosci. 2022 Jun 1;16:888814. doi: 10.3389/fnins.2022.888814 (PMC9198483; doi:10.3389/fnins.2022.888814)

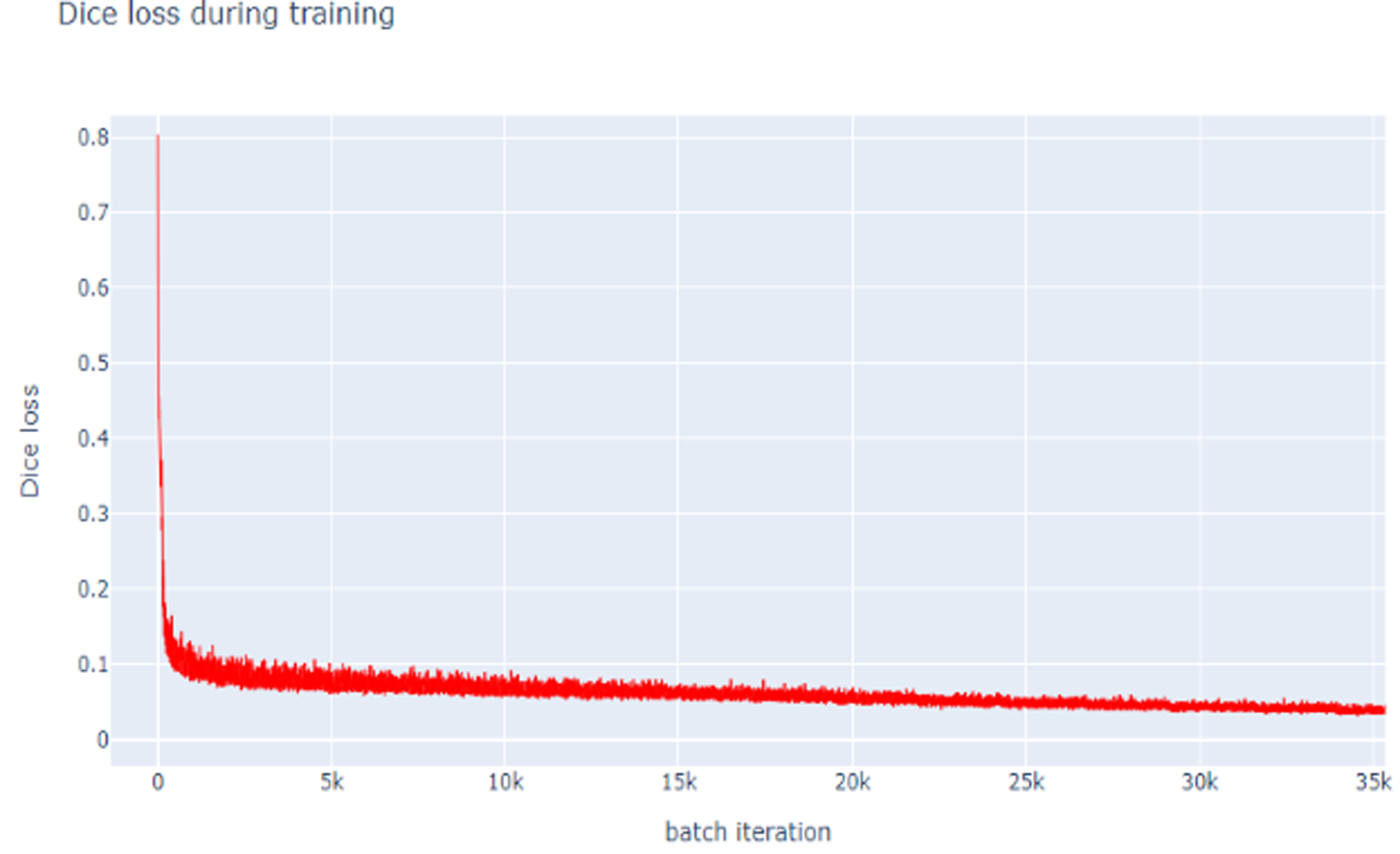

Supplement: Supplementary Figure 1 — The plot of convergence for training data. [file Image_1.TIF]

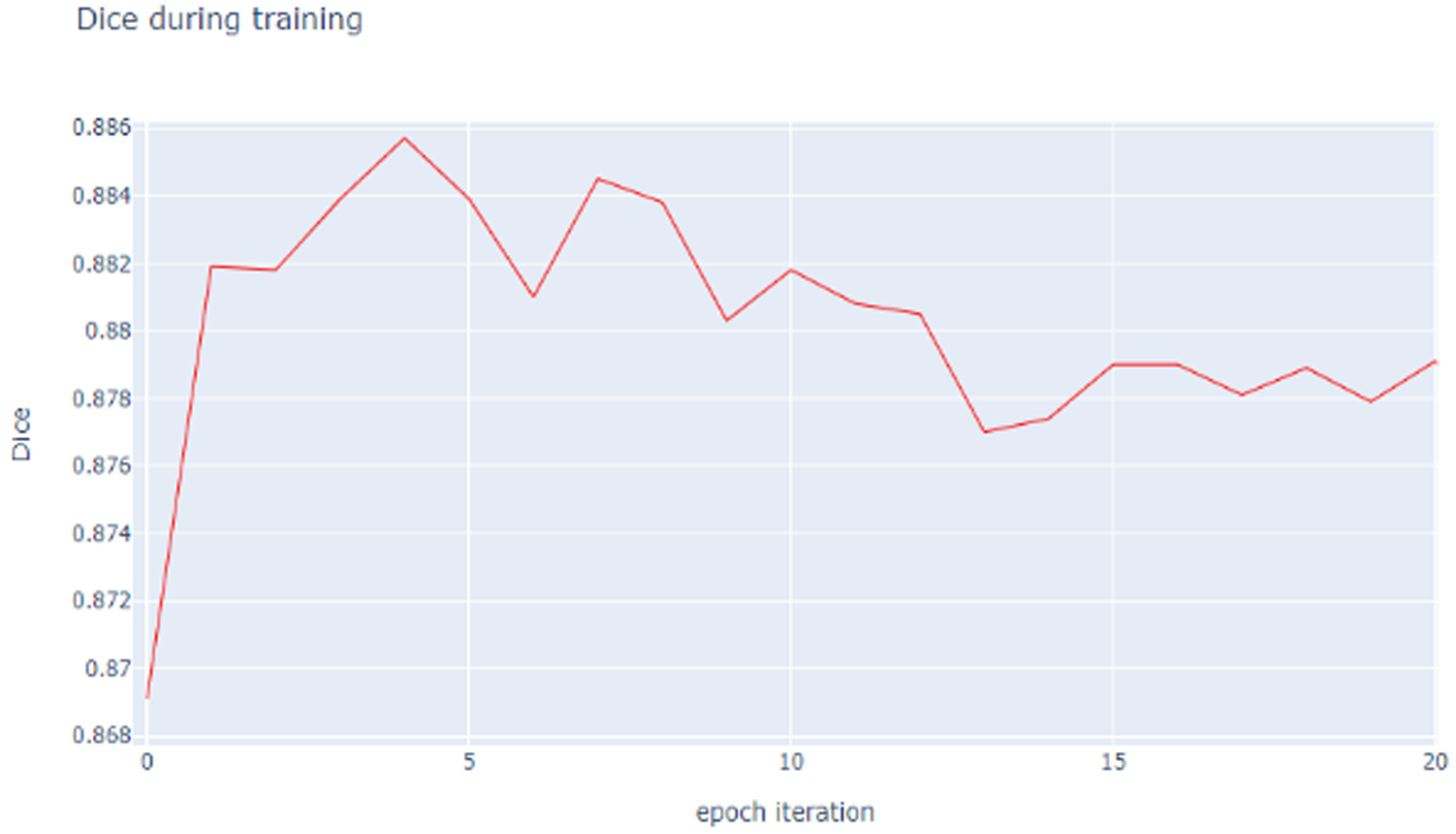

Supplement: Supplementary Figure 2 — The plot of convergence for validation data. [file Image_2.TIF]

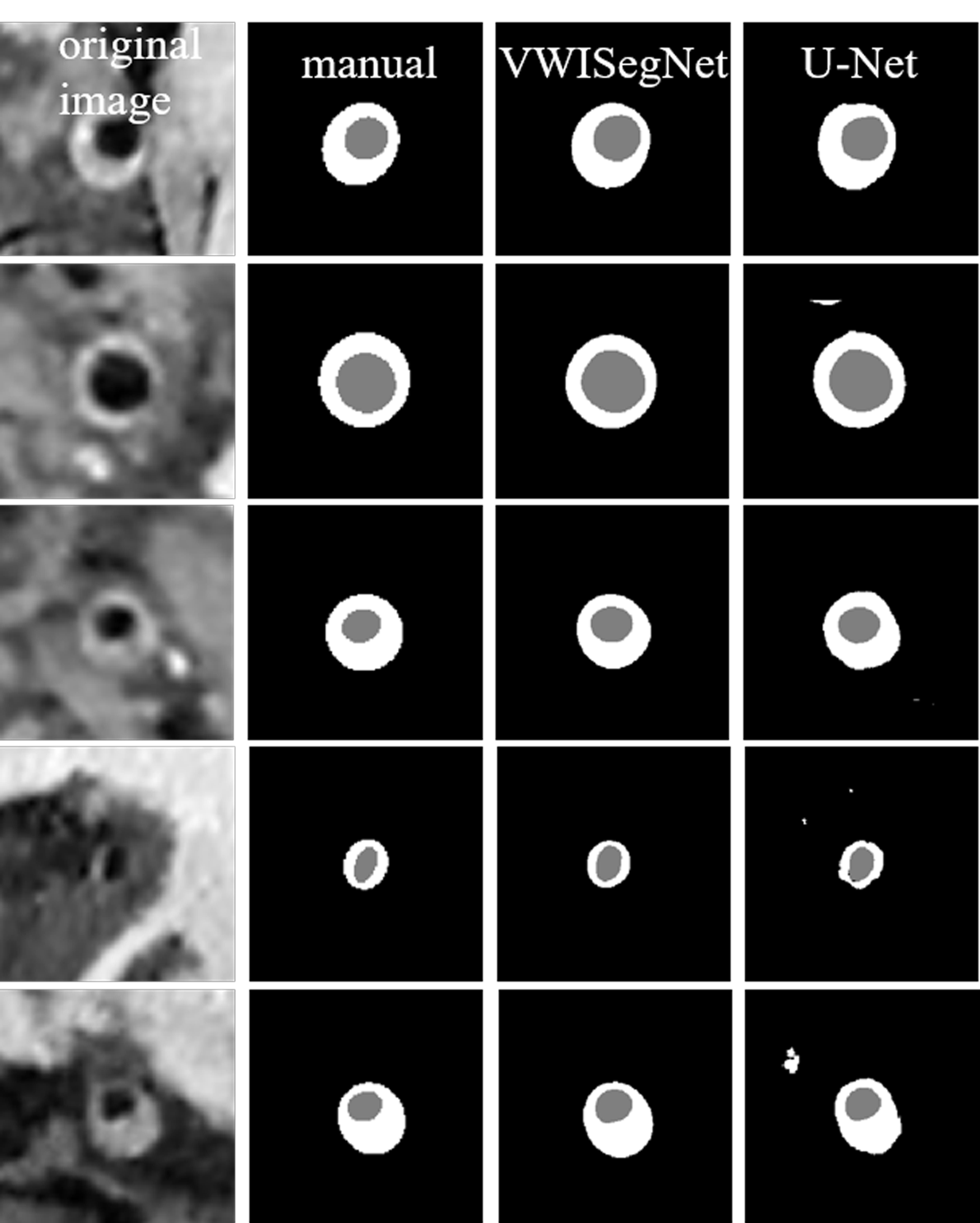

Supplement: Supplementary Figure 3 — The training convergence plot of U-Net. [file Image_3.TIF]

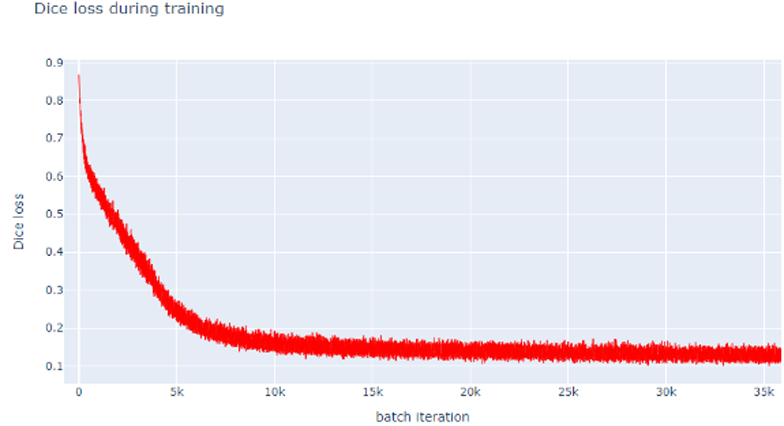

Supplement: Supplementary Figure 4 — The training convergence plot of Attention U-Net. [file Image_4.TIF]
